# Secular Changes in Widespread Pain Across Two Generations: The Framingham Heart Study

**DOI:** 10.1093/geroni/igaf122.072

**Published:** 2025-12-31

**Authors:** David Felson, Juan-Pablo Zertuche, Nene Ukonu, Michael LaValley, Margaret Clancy, Tuhina Neogi

**Affiliations:** Boston University Chobanian & Avedisian School of Medicine, Boston, Massachusetts, United States; Boston University Chobanian & Avedisian School of Medicine, Boston, Massachusetts, United States; Boston University Chobanian & Avedisian School of Medicine, Boston, Massachusetts, United States; Boston University Chobanian & Avedisian School of Medicine, Boston, Massachusetts, United States; Boston University Chobanian & Avedisian School of Medicine, Boston, Massachusetts, United States; Boston University Chobanian & Avedisian School of Medicine, Boston, Massachusetts, United States

## Abstract

Ten to eleven percent of adults have widespread musculoskeletal pain (WSP) with a higher prevalence in older persons.  It is unknown whether WSP prevalence has changed nor whether increased obesity or longevity especially of women could explain this change.  We studied these questions in 2 generations of the community-based Framingham Heart Study cohorts. We studied the original cohort of the Heart Study in 1992-1995 and Generation2, the offspring of the original cohort and their spouses in 2019-2021, at a similar age to the original cohort. A minoritized community sample (OMNI cohort) was also assessed in 2019-2021. Participants were asked whether they had joint pain on most days and, those saying yes used a homunculus to identify sites of pain. We then determined whether a participant met the WSP definition: pain on the left and right sides of the body; pain above and below the waist, and spinal or low back pain. We tallied the prevalence of WSP in each cohort, adjusting for confounders using logistic regression to test for cohort differences. The mean age of participants, mostly women, was in the 70s (table 1). Just over 14% of both original and OMNI cohorts reported WSP but 19-20% of Generation2 had WSP. Comparing Generation2 to the Original Cohort, WSP increased in prevalence (adjOR = 1.97 (95% CI: 1.49 - 2.61, p <.001). The prevalence of WSP has risen in older persons, an increase not explained by increasing pain with age, increased longevity of women nor increasing BMI.

